# Decision-making under ambiguity and risk and executive functions in Parkinson’s disease patients: A scoping review of the studies investigating the Iowa Gambling Task and the Game of Dice

**DOI:** 10.3758/s13415-023-01106-3

**Published:** 2023-05-17

**Authors:** Laura Colautti, Paola Iannello, Maria Caterina Silveri, Alessandro Antonietti

**Affiliations:** Department of Psychology, Catholic University of the Sacred Heart, Laura Colautti, Largo A. Gemelli, 1, 20123 Milan, Italy

**Keywords:** Parkinson’s disease, Decision making, Executive functions, Dopamine, Reward, Cognition

## Abstract

Evidence shows that patients affected by Parkinson’s disease (PD) display the tendency toward making risky choices. This is due, at least in part, to the pathophysiological characteristics of the disease that affects neural areas underlying decision making (DM), in which a pivotal role is played by nonmotor corticostriatal circuits and dopamine. Executive functions (EFs), which can be impaired by PD as well, may sustain optimal choices in DM processes. However, few studies have investigated whether EFs can support PD patients to make good decisions. Adopting the scoping review approach, the present article is designed to deepen the cognitive mechanisms of DM under conditions of ambiguity and risk (that are conditions common to everyday life decisions) in PD patients without impulse control disorders. We focused our attention on the Iowa Gambling Task and the Game of Dice Task, because they are the most commonly used and reliable tasks to assess DM under ambiguity and under risk, respectively, and analyzed the performances in such tasks and their relationships with EFs tests in PD patients. The analysis supported the relationships between EFs and DM performance, especially when a higher cognitive load is required to make optimal decisions, as it happens under conditions of risk. Possible knowledge gaps and further research directions are suggested to better understand DM mechanisms in PD sustaining patients’ cognitive functioning and preventing negative consequences in everyday life derived from suboptimal decisions.

## Introduction

Parkinson’s disease (PD) is the second most common neurodegenerative disease, with an incidence of approximately 1-2% in older adults (Chen et al., 2022). PD can be described as a slowly degenerative neurological disease, due to a loss of dopaminergic neurons in the substantia nigra pars compacta of the mesencephalon and an accumulation of the α-synuclein protein, which constitutes insoluble aggregates forming the basis of Lewy bodies. Alterations in the nigrostriatal circuit occur as well (Balestrino & Schapira, 2020; Zgaljardic et al., 2006). Progressing the disease, other brain areas are involved, such as cortical regions, in particular the prefrontal cortex (PFC) (Braak et al., 2003; Braak & Del Tredici, 2008; Ferrer, 2009), and the corticostriatal pathways. Focusing on the latter ones, they are composed of five different circuits receiving partially overlapping corticostriatal inputs and projecting to distinct striatal regions. They are the motor and the oculomotor circuits (that are usually categorized as motor circuits), the dorsolateral prefrontal, the (lateral) orbitofrontal, and the anterior cingulate circuits (usually classified as complex or non-motor circuits) (Alexander et al., 1986; Zgaljardic et al., 2006). Focusing on the nonmotor circuits, each of them underlies a specific prefrontal area and it is assumed to be linked with distinct cognitive and behavioral functions: Respectively, the dorsolateral prefrontal cortex (dlPFC) is thought to mediate cognitive functions, such as executive functions (EFs); The orbitofrontal cortex (OFC) has been linked to functions such as impulse control, decision making based on reinforcement and reward, mood regulation; The anterior cingulate cortex (ACC) is thought to be involved in control and attentional processes (Zgaljardic et al., 2003).

PD is mainly characterized by the presence of motor symptoms (e.g., bradykinesia, resting tremor, rigidity of limbs and trunk, and postural instability) (Balestrino & Schapira, 2020; Moustafa et al., 2016). Motor symptoms are associated with a broad spectrum of nonmotor disturbances as well, including behavioral, affective, and cognitive disorders (Pfeiffer, 2016). Among the cognitive abilities, attention, EFs (e.g., divided attention, set-shifting and flexibility, inhibition, working memory, planning, executive control), speed processing, visuospatial skills, and learning (Dujardin & Laurent, 2003; Pillon et al., 1993; Poletti & Bonuccelli, 2010) are mainly affected. PD patients also can display impairments in Theory of Mind (ToM), increasing in severity with the progression of the disease (Bora et al., 2015; Coundouris et al., 2020). ToM can be described as the capacity to attribute mental states to oneself and others, including emotions, intentions, desires, beliefs, and knowledge (Premack & Woodruff, 1978).

During the past 20 years, studies also have paid attention to possible impairments in value-based decision making (DM) in PD, which is characterized by making a choice through the attribution of subjective values to the different choice options (Rangel et al., 2008). On the basis of information available to make the decision, two conditions can be conceptualized: DM under ambiguity and DM under risk. In the previous condition, the probabilities of the occurrence of positive and negative consequences—associated with at least one of the possible choices—are unknown. In the latter condition, the probabilities of the occurrence of positive and negative consequences are known (Brand et al., 2007; Lauriola et al., 2007; Schultz et al., 2008) (for more details about DM, see Table 1).

**Table 1:** Brief general introduction to decision making

Definition	Making a decision generally means selecting, at least between two options, the one that can maximize the desirable consequences and minimize the possible costs (Rangel et al., 2008; Kim and Lee, 2011). DM is composed of multiple steps that can be influenced by affective aspects, such as motivation and emotions triggered by the contingent situation, which can affect the decisional process since its earlier steps (Rangel et al., 2008; Ryterska et al., 2014; Schiebener and Brand, 2015; Colautti et al., 2022). The first one is the representation of the decisional problem (identifying relevant information). Other steps include the evaluation of the options (assigning values and costs to them basing on information concerning the probability of occurrence of possible positive/negative outcomes and efforts needed), the execution of the required actions, the evaluation of the outcome (comparing it to the expected one), and, finally, learning from feedback (updating previous knowledge and subjective representations with the contingent outcome to optimize future choices).
Behavioral assessment	Usually, the tasks mainly adopted to assess DM under ambiguity and risk and collect behavioral responses involve monetary decisions (i.e., win or loss of a monetary amount) and strongly underlie the DM processes mentioned above, focusing on the evaluation of choice options and outcomes through the analysis of positive consequences (or rewards) and negative ones/costs (or losses). Specifically, tasks designed to assess DM under ambiguity mainly involve implicit rules for wins and losses associated to the different choice options (as it happens in the Iowa Gambling Task (Bechara, 2007; Bechara et al., 1994) or in the Balloon Analogue Risk Task (Lejuez et al., 2002). Conversely, tasks addressed to DM under risk present stable and explicit rules for wins and losses characterizing the different choice options (as it happens with the Game of Dice (Brand et al., 2005) or the Cambridge Gambling Task (Rogers et al., 1999)).
Assessment of the underlying processes	To deepen the cognitive processes underlying the behavioral responses recorded through DM tasks some studies also adopted other noninvasive techniques in association with the administered tasks. The ones most frequently adopted are neuroimaging techniques to investigate the neural activation underlying decisional processes (e.g., functional magnetic resonance imaging, positron emission tomography, electroencephalography) and the acquisition of autonomic data to explore emotional psychophysiological responses before making a choice or after receiving feedback (e.g., heart rate variability, electrodermal activity) (Forte et al., 2021; Hartmann et al., 2020).

DM, decision making

Reviews of literature showed that patients affected by PD generally present a tendency toward making more risky choices under both conditions of ambiguity and risk compared with healthy controls (HCs), although results are controversial when exploring possible significant differences between PD patients and HCs in DM tasks (Colautti et al., 2021; Evens et al., 2016; Kjær et al., 2018). Conversely, it appears crucial to acquire more knowledge regarding patients’ trend toward making risky choices and the underlying mechanisms, because in everyday life it may lead to behavioral disturbances, such as impulse control behaviors (ICBs) or more severe forms of impulse control disorders (ICDs) (for a definition, see Weintraub et al., 2015), that can give rise to negative consequences for patients’ care process and quality of life (Baig et al., 2019; Drew et al., 2020; Stenberg, 2016).

To better understand such an issue, with the further goal to promote optimal levels of cognitive functioning and wellbeing in PD patients, the present scoping review was designed to delve into the cognitive mechanisms of DM in PD patients, analyzing the relationship between DM under conditions of ambiguity and risk and EFs, which are pivotal in the decisional process (Brand et al., 2006; Del Missier et al., 2010; Schiebener and Brand, 2015; Colautti et al., 2022), the underlying neural substrates, and their possible involvement in PD. First, a brief overview of the DM process in PD is reported, followed by an analysis of the underlying neural mechanisms. Afterward, results derived from a scoping review analysis are provided and discussed to deepen the relationship between DM and EFs in PD patients, highlighting possible outstanding questions about the decisional processes in PD and suggesting directions for new research.

### What is known about decision making in Parkinson’s disease

There is a broad consensus about the presence of a preference toward risky choices in PD patients—probably due to an impairment in anticipating the unrewarding consequences or to an insensitiveness to punishment—at least in part due to the involvement of brain structures supporting decisional processes and affected by the disease (Colautti et al., 2021; Kobayakawa et al., 2010). Conversely, results are mixed when behavioral responses are statistically analyzed to investigate possible overt differences between patients and healthy controls in DM tasks, probably due to the variability of the processes involved. In fact, it is assumed that PD affects selectively some steps of DM rather than leading to a general and unspecific impairment (Ryterska et al., 2014). Whilst some cognitive processes of DM (such as recognizing and representing the decisional situation) are mostly spared, others are crucially impaired. One of these is the evaluation of choice options, in which it is required to make a “cost-benefit analysis” according to personal goals, to set values in terms of positive or negative outcomes to the possible options, and to anticipate consequences. Other processes that are affected are the evaluation of the outcome and learning from feedback, essential to make further optimal choices. Hence, in those decisional tasks where these processes cover a pivotal role, as it happens at different levels in situations under ambiguity and risk, the behavioral tendency of patients to make risky and suboptimal choices can be clearer (Foerde & Shohamy, 2011; Ryterska et al., 2014).

Possible explications for such a behavior can be inferred from the type of neural structures affected in PD, the progressive loss of dopamine in the disease evolution, and the dopaminergic therapy (Kjær et al., 2018; Kobayashi et al., 2019). The progressive decrease in dopamine in PD reduces the functionality of corticostriatal circuits. Specifically, two of them are assumed to be more related to cognitive impairments: the dorsolateral circuit—counting dlPFC, striatum (dorsolateral caudate nucleus), globus pallidus, and thalamus—and the orbitofrontal circuit—counting OFC, striatum (ventromedial caudate nucleus), globus pallidus, and the thalamus (Poletti & Bonuccelli, 2013; Zgaljardic et al., 2003). In both circuits, two loops connect the striatum to the PFC: the direct excitatory loop (Go pathway of the BG) and the indirect inhibitory loop (No-go pathway), which modulate motivation and reward processes. Rewards and punishments induce respectively phasic dopamine bursts or dips (Kravitz et al., 2012; Volkow & Morales, 2015). Bursts arouse the Go pathway (easing a cortical response—reward processing), whereas dips act on the No-go pathway (inhibiting actions from being executed to contrast negative consequences—punishment learning) (Argyelan et al., 2018). In this way, the depletion of dopamine, as it happens in PD, may promote No-go pathway activity, increasing punishment learning. Whilst increased levels of dopamine, as it may happen with dopaminergic drugs intakes, can elicit the opposite result, promoting the Go pathway that can both increase sensitivity to reward (Cools et al., 2022; Hikida et al., 2010) and prevent dopamine dips (Poletti & Bonuccelli, 2013). Studies exploring dopamine replacement therapy withdrawals showed a decreased sensitivity toward punishment in pharmacological “on” conditions rather than “off” conditions and a decreased neural response to negative feedback as well (Argyelan et al., 2018; McCoy et al., 2019). Therefore, this condition may sustain greater processing of reward than punishment (Benussi et al., 2017), which can explain both the patients’ trend to be more focused on positive consequences (regardless of possible higher negative ones) and the possible behavioral impulsivity displayed in making hasty choices in everyday life situations (Kjær et al., 2018). In fact, the imbalance in learning from punishment and reward together with the higher sensitivity to the latter one can lead patients to develop risky behaviors, with detrimental personal, social, financial, and medical consequences for themselves and their families (Drew et al., 2020).

Moreover, the tendency toward making risky choices and the insensitiveness to unrewarding consequences detected in PD patients have been explained by other mechanisms focused on the possible effect of dopamine on the ventral striatum. Specifically, during the onset stages of PD, the decrease in dopamine mainly involves the dorsolateral circuit affecting the functioning of EFs, whereas the orbitofrontal circuit (mainly underlying emotion-based representations, processing of reward, implicit and probabilistic reversal learning) is usually affected in later stages (Cools et al., 2022; Poletti & Bonuccelli, 2013). According to the dopamine overdose hypothesis (for more details, see Gotham et al., 1986; Cools et al., 2022), the administration of dopaminergic drugs may produce differential cognitive effects on such frontostriatal circuits, improving cognitive functions, such as EFs, mainly relying on the dorsolateral circuit, while affecting those related to the more spared ventral striatum and orbitofrontal circuit, which are involved in DM under ambiguity and risk, by “overdosing” them. This hypothesis is in line with studies investigating the effects of dopaminergic drugs withdrawals, where the pharmaceutical “off” condition shows increasing difficulties in tasks related to components of the dorsolateral circuit, such as tasks requiring set-shifting, but improvements in tasks related to the orbitofrontal circuit, such as tasks requiring reversal learning (Cools et al., 2001; Jahanshahi et al., 2010). Thus, the involvement of neural structures pivotal for learning contingencies under ambiguous situations, cost-benefit analysis, reward anticipation, reward and risk processing, such as OFC and ventral striatum (Colautti et al., 2021; Pascucci et al., 2017; Ryterska et al., 2014; Seymour et al., 2007; Zha et al., 2022), can explain, at least in part, the selective impairments in DM steps that are involved in risky and ambiguous conditions.

### The important role of executive functions in decision making

DM implies many cognitive functions (e.g., general cognitive abilities, long-term memory, numerical and probability processing), among which EFs appear to be crucial (Schiebener and Brand, 2015; Colautti et al., 2022). Specifically, decision makers are required to inhibit the drive to be attracted by impulsive choices planning a strategy that allows them to achieve long-term wins rather than making choices characterized by possible higher immediate wins but also long-term higher losses (Colombo et al., 2020). Furthermore, decision makers have to compare possible choices’ outcomes holding in mind relevant data, remember the outcomes of previous choices, update representations of subjective values of the possible options, and shift their future decisions according to the available data and the updated representations (Colautti et al., 2021). These cognitive operations mainly involve flexibility, inhibition, planning, and working memory, which are included in EFs and can be impaired in PD patients. As shown by recent studies focusing on healthy adults (Colautti et al., 2022; Damme et al., 2019), the degree of involvement of EFs in DM depends on some elements, such as the nature of the decisional problem, the kind of information available, the affective components triggered by the situation, and the level of the decision maker’s cognitive functioning.

Data from neuroimaging studies highlighted overlaps between brain areas implied in DM tasks and in EFs tests, where a crucial role is played by the frontostriatal loops (in which PFC and basal ganglia (BG) are pivotal) and dopaminergic pathways. They are considered fundamental in motivation, reward processing, and learning (Brand et al., 2006; Costello et al., 2021; Euteneuer et al., 2009), as well as in EFs, such as working memory and set-shifting (Giehl et al., 2019; Kehagia et al., 2013; Monchi et al., 2006). Specifically, BG, ventromedial and ventrolateral PFC (vmPFC and vlPFC), and OFC are assumed to be involved in processing of feedback, emotionally connoted stimuli, and in the ability to anticipate future consequences (Gleichgerrcht et al., 2010; Pascucci et al., 2017; Rolls, 2000). In addition, the dlPFC and medial PFC (mPFC), as well as the ACC, may be engaged in risk/reward processing, error-detection ability, and cognitive control (Kondo et al., 2004; McCormick et al., 2019; Ramchandran et al., 2020).

Nevertheless, considering PD population and its pathophysiological features, and in particular (a) the patients’ risk-taking tendency described in literature, (b) the possible effects of dopamine on neural structures underlying EFs functioning and DM under ambiguity and risk, and (c) the crucial contribution of EFs on the decisional process, it appears important to focus on the relationship between DM and EFs to clarify those EF cognitive abilities that can support DM in patients or that, if impaired, may contribute to DM alterations.

## Goals

According to the goals of a scoping review (Munn et al., 2018), the present article wants to provide the emergent results of a thorough analysis of the studies in literature about DM under ambiguity and risk in PD patients, exploring the relationship between decisional performance and EFs, highlighting possible gaps and further research questions. To date, no literature reviews have systematically investigated this relationship in PD patients through a comprehensive overview of the issue.

To investigate decisional competencies, we decided to focus principally on the Iowa Gambling Task (IGT; Bechara et al., 1994; Bechara, 2007) and the Game of Dice Task (GDT; Brand et al., 2005), which assess DM under ambiguity and under risk, respectively (see Table 2 for a detailed description of the tasks). We made this decision since they are considered the most commonly used and reliable tasks by the literature to address situations of ambiguity and risk (Buelow, 2015).

**Table 2:** Description of two decisional tasks: the Iowa Gambling Task and the Game of Dice Task

Task	DM condition assessed	Brief description	Parameters recorded
Iowa Gambling Task – IGT (Bechara, 2007; Bechara et al., 1994)	DM under ambiguity	The decision maker has to increase as much as possible an initial monetary capital, by choosing a card per trial from four decks (A, B, C, D) within 100 total trials. Each deck is designed for having either monetary wins or losses with different probabilities and frequencies of occurrence. Usually, decks A and B are disadvantageous, as they are characterized by high wins (or rewards) but also higher losses (or punishments), leading the decision maker to a final overall loss. Conversely, decks C and D are advantageous, because they are characterized by small wins and losses, leading to a final overall gain. It is assumed that two main processes are involved in the task. In the first part of the task the decision maker does not know the probability of win and loss for each deck; So, the early decisions are near to the chance level (DM under predominantly ambiguity). During further trials, a representation of the advantageous and disadvantageous decks begins to arise by the occurrence of win-and-loss feedback related to each deck (DM under risk).	- Total amount of money (either positive or negative) at the end of the 100 trials; - Total net score (advantageous minus disadvantageous selections throughout the 100 trials); - Net score by dividing the performance into blocks of equal trials (e.g., two blocks composed of 50 trials, or five blocks of 20 trials); - Number of selections from each deck.
Game of Dice Task – GDT (Brand et al., 2005)	DM under risk	The decision maker has to increase an initial monetary capital by selecting which face(s) of a die will come out after each throw. There are in total 18 trials, which correspond to the number of throws of the die. To decide, the decision maker has to select among a single number (one face) or a combination of two to four numbers of a die (two to four faces) after every throw. The specific win-and-loss amount and the occurrence probability are explicitly expressed. As well, the rules are stable throughout the task. To make optimal decisions, both the possible win amount and the probability of occurrence have to be considered (e.g., if the decision maker selects a single number and the chosen number occurs (1:6 chance), he/she wins the highest monetary amount. If the decision maker selects a combination of two numbers, the amount is a little lower, but the probability to win is a little higher (2:6 chance). So on for a combination of three numbers, until the decision maker selects a combination of four numbers, where the win value is the lowest, but the probability of occurrence is the highest (4:6 chance)). Accordingly, selections of one or two numbers are categorized as disadvantageous/risky, whereas the selections of three or four numbers are categorized as advantageous/safe. To develop a successful and goal-oriented strategy to complete the task, the decision maker must examine all data available.	- Net score (advantageous minus disadvantageous choices); - Frequency of choosing risky/safe options.

DM, decision making

## Method

The review was led according to the Preferred Reporting Items for Systematic Reviews and Meta-Analysis Extension for Scoping Reviews (PRISMAScR) (Tricco et al., 2018). A registered protocol is not required for scoping review (Munn et al., 2018).

The methodology of the review was designed according to the five-stage framework (Arksey & O’Malley, 2005) to provide transparency and to increase the reliability of the findings and the replicability of search strategies. Below, the five stages are reported in detail.

### Identifying the research question (Stage 1)

The present scoping review was led to answer the following questions: Is there a relationship between EFs and DM under ambiguity in PD patients? Is there a relationship between EFs and DM under risk in PD patients?

### Identifying relevant studies (Stage 2)

We examined studies that investigate DM under ambiguity and risk through the IGT and the GDT, which are the most reliable tasks investigating these constructs (see Goals paragraph for further details).

The last search update was made in May 2022. It encompassed articles published since 2000 in peer-reviewed journals indexed in Scopus, PubMed, and PsycINFO. The keywords entered were “Iowa Gambling Task AND (executive functions) AND (Parkinson’s Disease),” “Game of Dice Task AND (executive functions) AND (Parkinson’s Disease).” After Stage 3, the references of the selected studies were checked to include other possible eligible studies.

Then, the following inclusion criteria were adopted, ensuring comparisons among the studies to be analyzed: 1) studies that recruited samples of patients affected by PD in treatment with dopaminergic replacement therapies, which is the most common clinical condition for PD patients; 2) studies that assessed EFs through the administration of validated instruments; 3) studies that did not present a modified version of the IGT nor the GDT.

The exclusion criteria were: 1) studies in which patients are affected by an atypical parkinsonism (i.e., progressive supranuclear palsy, multiple system atrophy, corticobasal degeneration); 2) studies in which patients presented a diagnosis of ICD; 3) samples of subjects affected by psychiatric comorbidity (e.g., major depression, obsessive-compulsive behaviors, schizophrenia); 4) studies in which patients received deep brain stimulation; 5) studies in which participants underwent interventions to foster cognitive functioning (e.g., cognitive training, transcranial electric stimulation techniques); 6) book chapters.

### Study selection (Stage 3)

**Fig. 1:**
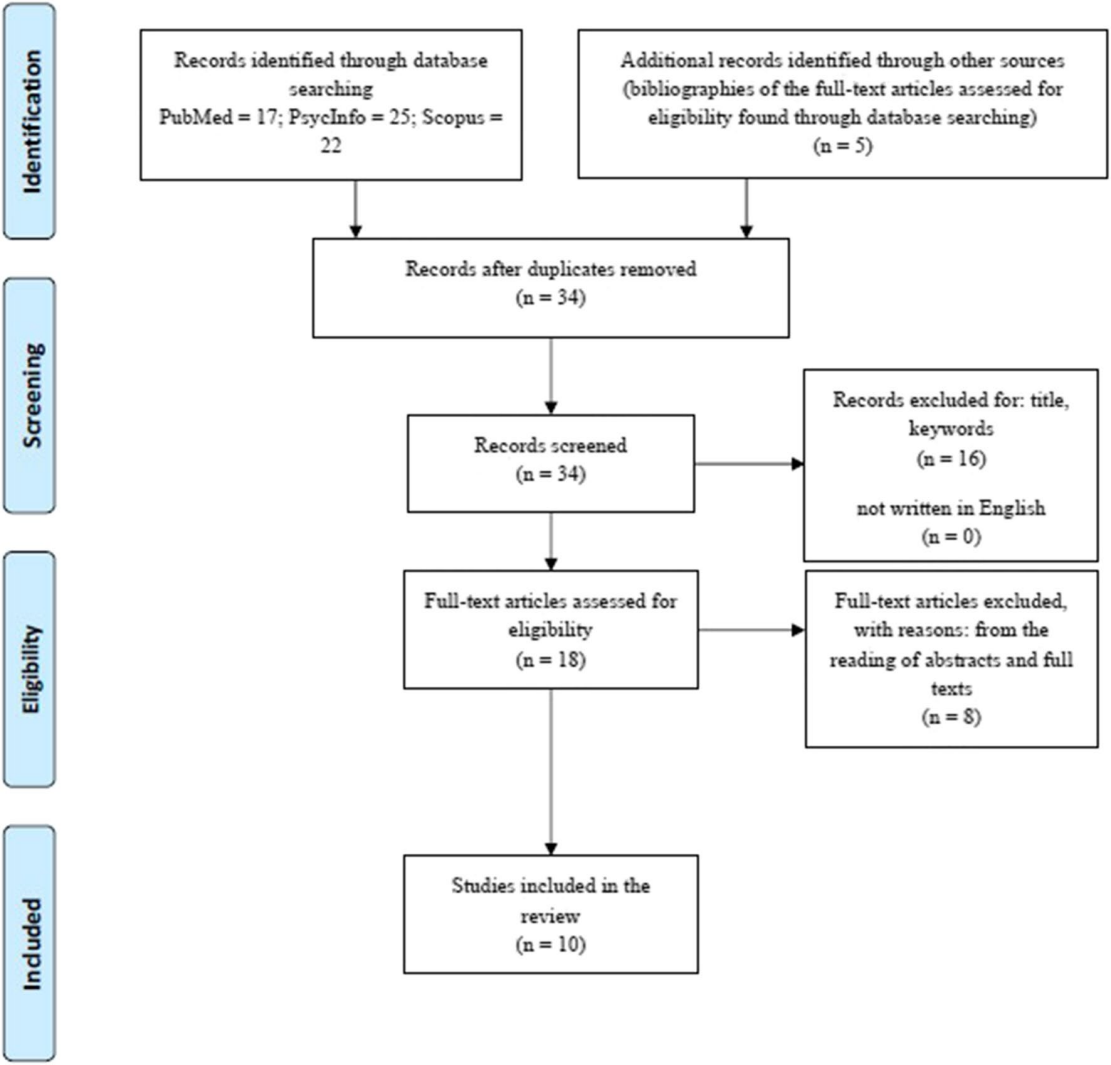
PRISMA flow diagram for the considered studies

Relevant articles were screened by one author (LC): first by title, keywords, and language used, and then by reading abstracts and full texts. The PRISMA Statement (Moher et al., 2009) was followed for the selection of the studies (Fig. 1). Possible doubts concerning the inclusion of the studies were analyzed by two other authors (AA and PI).

### Charting the data (Stage 4)

A form was drafted to determine which variables to examine. When possible, the extraction of data followed Arksey and O’Malley’s (2005) recommendations to ensure comparisons between studies. Data reported concern authors and year of publication, countries in which the studies were led, samples characteristics (i.e., size of the sample, age and education information, the duration and the severity of the disease, “on”/“off” conditions in which patients were tested—considering the timing of dopaminergic drugs intake; Levodopa-equivalent daily dose – LEDD), parameters of the decisional tasks, assessed EFs and other cognitive functions and the tools used, and main results.

### Collating, summarizing, and reporting the results (Stage 5)

To provide a narrative report of the results, an analytic framework was considered following the PRISMA guidelines – extension for scoping review (Tricco et al., 2018).

## Results

### Characteristics of the selected studies

A total of ten studies had been selected (Fig. 1), among which nine had administered the IGT (Euteneuer et al., 2009; Gescheidt et al., 2012; Ibarretxe-Bilbao et al., 2009; Kobayakawa et al., 2008; Kobayakawa et al., 2010; Mimura et al., 2006; Pagonabarraga et al., 2007; Perretta et al., 2005; Xi et al., 2015) and three the GDT (Brand et al., 2004; Euteneuer et al., 2009; Xi et al., 2015). In two studies, both the IGT and the GDT had been administered to the same samples (Euteneuer et al., 2009; Xi et al., 2015).

### Investigating the IGT: DM performance and its relationship with EFs

Among the studies that investigated the IGT, three were performed in Japan, one in China, one in Canada, and four in Europe (Spain, Germany, and Czech Republic).

Patients’ lowest mean age was 50.32 years for early-onset PD (namely, patients who received a diagnosis at age ≤ 45 years) (Gescheidt et al., 2012) and the highest were 77.7 and 72.4 years for patients in the 1° - 2.5° and 3° - 4° stage of diseases progression respectively in the Hoehn and Yahr scale (Hoehn & Yahr, 1967) (Perretta et al., 2005). The highest mean disease duration was 11.3 years (Gescheidt et al., 2012).

In all the studies considered, PD patients had no major cognitive impairments, mainly examined through the Mini-Mental State Examination (MMSE; Folstein et al., 1975) or other validated clinical tools (Table 4). Parameters used by authors to investigate the decisional performance generally confirmed the tendency of patients to make more suboptimal and risky choices than HCs (namely, preferring decks connoted by higher wins but also higher losses). In five of six studies, PD patients presented a significantly lower total net score compared with HCs (Euteneuer et al., 2009; Gescheidt et al., 2012; Ibarretxe-Bilbao et al., 2009; Kobayakawa et al., 2008, 2010; Pagonabarraga et al., 2007). Only in Euteneuer et al. (2009), no significant differences emerged, even though patients’ overall net score was lower than the HCs’ one. Another parameter used by authors relies on dividing the task into blocks (generally composed of five blocks each containing 20 trials or two blocks each encompassing 50 trials). In most of the studies, the PD group performed lower than the HC one and the difference appeared significant from the second or the third block out of five (Gescheidt et al., 2012; Ibarretxe-Bilbao et al., 2009; Kobayakawa et al., 2008, 2010; Mimura et al., 2006; Xi et al., 2015). When authors explored the total earned, it was significantly lower in the patients’ group than in the HCs (Kobayakawa et al., 2008, 2010; Mimura et al., 2006). Other parameters were used by single studies, revealing that the PD group made a lower number of advantageous choices (Perretta et al., 2005; Pagonabarraga et a., 2007; Gescheidt et al., 2012; Xi et al., 2015; see Table 4 for more details).

Relationships between the IGT and EFs emerged in two of nine studies (Ibarretxe-Bilbao et al., 2009; Pagonabarraga et al., 2007). Specifically, Pagonabarraga et al. (2007) found negative correlations between decisional performance and verbal fluencies (both phonemic and semantic). In this sample, the better were the performances in the decisional tasks, the worse were the abilities involved in verbal fluency tasks that require processing verbal information, cognitive flexibility, and rule monitoring (Strauss et al., 2006). Furthermore, negative correlations also emerged with global cognitive functioning and the free recall of a word list. Thus, the better patients performed in the decisional task, the worse they then performed in the cognitive tests. Such results reveal an opposite trend with respect to what emerged from other studies examining decisional processes both in PD patients and in other pathological conditions as well as in healthy samples (Fein et al., 2007; Labudda et al., 2009; Shurman et al., 2005). These results should be treated with caution (Colautti et al., 2021) and therefore will not be considered further.

In the other study, a positive relationship between the IGT performance and the Digit span backward was reported (Ibarretxe-Bilbao et al., 2009). So, the more PD patients made advantageous choices, the more they showed higher working memory abilities. Furthermore, authors highlighted a positive correlation between the IGT and the recognition of emotions.

The other selected studies failed to find significant relationships between the decisional performance and, at least, one of the EFs tests. Otherwise, Mimura et al. (2006) reported a positive correlation between the IGT and the attribution of mental states to other people and recognizing emotions, consistently to Ibarretxe-Bilbao et al.’ (2009) results. Xi et al. (2015) found similar results. Euteneuer et al. (2009) found only a positive correlation between the IGT and the GDT providing a relationship with the ability to decide under conditions of risk. Perretta et al. (2005) found no correlations between the IGT and cognitive abilities, but the authors pointed out a positive correlation between the decisional task and a self-report questionnaire assessing depression in early PD patients, highlighting that this emotional state (that in the sample was in the “minimal depression range”) can support advantageous decisions. Similar results emerged also in Kobayakawa et al. (2008). Finally, only Euteneuer et al. (2009) did not find any correlation between the IGT and other investigated measures of EF.

Furthermore, concerning possible relationships between DM performance and both the dopaminergic replacement therapy dosage and patients’ clinical features (e.g., the duration of the illness, the onset age, the severity of PD), only five studies explicitly stated to have investigated them, finding no significant results (Euteneuer et al., 2009; Gescheidt et al., 2012; Ibarretxe-Bilbao et al., 2009; Kobayakawa et al., 2008, 2010).

### Investigating the GDT: DM performance and its relationship with EFs

Among the studies investigating the GDT, one of them was performed in China and two in Germany. Patients’ lowest mean age was 60.73 years (Xi et al., 2015) and the highest was 67.6 years (Euteneuer et al., 2009) (Table 3). The highest mean disease duration was 8.84 years (Brand et al., 2004).

**Table 3:** Features of the samples

	Country	Size of the sample	Age (yr): mean (SD)	Education (yr): mean (SD)	Disease duration: mean (SD)	UPDRS III: mean (SD)	Hoehn and Yahr:	LEDD (mg): mean (SD)	Assessment condition
*Iowa Gambling Task*									
Perretta et al., 2005	Canada	Early PD: 16 Later PD: 16 HC: 19	Early PD: 72.4 (±2.3) Later PD: 77.7 (±1.5) HC: 72.6 (±1.9)	Early PD: 14.6 (±0.7) Later PD: 14.1 (±1.1) HC: 14.3 (±0.7)		Early PD: 11.3 (±1.1) Later PD: 27.2 (±1.3)	Early PD: 2.1 (±0.1) Later PD: 3.3 (±0.1)		On
Mimura et al., 2006	Japan	PD: 18 HC: 40	PD: 68.9 (±7) HC: matched to PD				2-3 (stages)		On
Pagonabarraga et al., 2007	Spain	PD: 35 HC: 31	PD: 67.2 (±8) HC: 70.2 ±10)	PD: 11.9 (±5) HC: 9.9 (±4)		21.2 (±8)	2.2 (±0.6)	870 (±495)	On
Kobayakawa et al., 2008	Japan	PD: 34 HC: 21	PD: 69.9 (±8.9) HC: 67.6 (±6.9)	PD: 13.2 (±2.7) HC: 14.4 (±2.4)	6.4 (±3.4) yr		1.52 (±0.75)	391 (±213)	On
Euteneuer et al., 2009	Germany	PD: 21 HC: 23	PD: 67.6 (±7.31) HC: 64.4 (±8.56)	PD: 11.1 (±1.89) HC: 11.7 (±1.84)	85.7 (±72.7) mo	17.7 (±9.2)	2.5 (median)	487.69 (±317.18)	
Ibarretxe-Bilbao et al., 2009	Spain	PD: 24 HC: 24	PD: 56.13 (±8.5) HC: 57.58 (±8.9)	PD: 10.96 (±5.4) HC: 13 (±3.8)	3.06 (±1.6) yr	14.67 (±3.5)	1.73 (±0.4)	299.58 (±321.1)	
Kobayakawa et al., 2010	Japan	PD: 14 HC: 22	PD: 68.9 (8) HC: 67.6 (6.9)	PD: 14.8 (±4.1) HC: 14.4 (±2.4)	5.6 (±2.7) yr		1.4 (±0.6)	476.9 (±184.1)	On
Gescheidt et al., 2012	Czech Republic	PD: 19 HC: 20	PD: 50.32 (±8.74) HC: 49.95 (±9.03)		11.3 (±6.4) yr	14.6 (±8.7)	1.7 (±0.57)		On
Xi et al., 2015	China	PD: 15 HC: 15	PD: 60.73 (±11.79) HC: 56.33 (±14.59)	PD: 8.93 (±2.89) HC: 9.27 (±3.08)	4.33 (±5.05) yr	15.87 (±8.96)	1.97 (±0.67)	254.58 (±132.2)	
*Game of Dice Task*									
Brand et al., 2004	Germany	PD: 20 HC: 20	PD: 66.85 (±9.68) HC: 64 (±7.25)	PD: 9.1 (±1.15) HC: 9.8 (±1.57)	106.05 (±81.11) mo	47.25 (±20.42)	3 (median)		
Euteneuer et al., 2009	Germany	PD: 21 HC: 23	PD: 67.6 (±7.31) HC: 64.4 (±8.56)	PD: 11.1 (±1.89) HC: 11.7 (±1.84)	85.7 (±72.7) mo	17.7 (±9.2)	2.5 (median)	487.69 (±317.18)	
Xi et al., 2015	China	PD: 15 HC: 15	PD: 60.73 (±11.79) HC: 56.33 (±14.59)	PD: 8.93 (±2.89) HC: 9.27 (±3.08)	4.33 (±5.05) yr	15.87 (±8.96)	1.97 (±0.67)	254.58 (±132.2)	

blank: not reported

HC, healthy control group; LEDD, Levodopa-equivalent daily dose; mo, months; PD, Parkinson’s disease group; UPDRS III, Unified Parkinson’s disease Rating Scale – motor section; yr, years

In all the studies considered, PD patients had no major cognitive impairments (mainly examined through the MMSE or other validated clinical tools) (Table 4). In all of the studies, PD samples performed more poorly than HCs in the GDT, making more suboptimal decisions by choosing more often one or two numbers of the die instead of three or four numbers and earning a lower monetary amount. In line with literature, patients preferred riskier options, characterized by reduced probabilities to achieve higher wins regardless of a higher probability of substantial losses (as it happens to choose a single number or a combination of two numbers of the die).

**Table 4:** Assessment and main results of the studies

	Decisional task parameters used for the analysis	EFs	Other cognitive functions	Assessment	Main findings
Iowa Gambling Task					
Perretta et al., 2005	Mean of card selections from the advantageous decks (blocks of 10 trials)	a) frontal lobe functioning	b) probabilistic learning c) depression d) global cognitive functioning	a) WCST, Stroop test b) PCL c) BDI d) MMSE	PD groups performed worse than HCs in the IGT. In early PD group, there was a positive correlation between IGT and depression (r = 0.51, *p* < 0.05).
Mimura et al., 2006	Total amount of money earned, Net score (two blocks composed of 50 trials)	a) set shifting b) planning c) inhibition d) verbal fluency and mental flexibility	e) ToM f) depression g) global cognitive functioning	a) WCST b) Maze-tracing c) CWIT d) Verbal fluency (phonemic and semantic) e) RMET f) SDS g) MMSE	PD group gained a lower total amount and a lower net score than the HC group in the IGT. The IGT score was correlated with ToM (r = 0.59, *p* < 0.01).
Pagonabarraga et al., 2007	Net score (total)	a) attention and EFs	b) memory c) global cognitive functioning	a) Stroop test (errors, time), Digit span (forward and backward), Verbal fluencies (phonemic and semantic) b) RAVLT c) MMSE, MDRS	PD group displayed a lower performance than HC group in the IGT. In PD patients, the IGT was correlated with MDRS (ρ = −0.56, *p* = 0.001), phonemic verbal fluencies (ρ = −0.62, *p* = 0.009) and semantic ones (ρ = −0.42, *p* = 0.01), and memory delayed recall (ρ = −0.56, *p* = 0.001).
Kobayakawa et al., 2008	Net score (total), Net score (5 blocks composed of 20 trials)	a) EFs	b) visuoperceptual abilities c)visual memory d) short term memory e) depression f) global cognitive functioning	a) WCST b) Rey-Osterrieth complex figure copy c) Rey-Osterrieth complex figure delay d) Digit span (forward and backward) e) SDS f) MMSE	PD group displayed a worse performance than HC group from the IGT second block, earning a lower total amount. Only a correlation emerged between the IGT and depression (ρ = −0.41, *p* < 0.05).
Euteneuer et al., 2009	Net score (total), Net score (5 blocks composed of 20 trials)	a) categorization, set shifting, flexibility, b) flexibility, rule monitoring, information processing c) working memory	d) ToM e) reasoning f) global cognitive functioning g) depression h) DM under risk	a) MCST b) Verbal fluencies (phonemic and semantic) c) Digit span backward d) RMET e) Reasoning subtest f) MMSE g) BDI h) GDT	PD group showed lower results in the IGT than HCs, but the difference was not significant. Otherwise, both groups displayed improvements after the first blocks. The IGT was correlated only with depression (r = 0.47, *p* = 0.033).
Ibarretxe-Bilbao et al., 2009	Net score (total), Net score (5 blocks composed of 20 trials)	a) working memory	b) sustained attention c) premorbid intelligence quotient d) recognition of facial emotion expressions	a) Digit span backward b) CPT II c) Vocabulary subtest d) Ekman 60 faces test	From the third block of the IGT, PD group presented a lower performance than HC group. The IGT was correlated with working memory (ρ = 0.48, *p* < 0.01) and the ability to recognize facial emotion expressions (ρ = 0.41, *p* = 0.02).
Kobayakawa et al., 2010	Net score (total), Net score (5 blocks composed of 20 trials)	a) EFs	b) visuoperceptual abilities c)visual memory d) short term memory e) depression f) global cognitive functioning	a) WCST b) Rey-Osterrieth complex figure copy c) Rey-Osterrieth complex figure delay d) Digit span (forward and backward) e) SDS f) MMSE	PD group earned lower gains than HC group. In the third and fifth blocks of the IGT, PD group scored significantly lower than HC group. There was no correlation between the IGT and the other abilities.
Gescheidt et al., 2012	Net score (total), Shift frequencies between the decks	a) EFs b) inhibition	c) global cognitive functioning	a) Tower of London b) Stroop test c) Matrix reasoning	PD group displayed a lower performance than HC group in the IGT. PD group shifted more frequently between advantageous/disadvantageous decks, while HC group made more advantageous selections. There was no correlation between the IGT and EFs in PD group.
Xi et al., 2015	Net score (5 blocks composed of 20 trials), Number of selections from advantageous/disadvantageous decks	a) frontal functions b) inhibitory control c) EFs	d) short term memory e) verbal memory f) visual perceptual function g) ToM h) DM under risk i) depression l) global cognitive functioning	a) Verbal fluency (semantic) b) Stroop test c) Digit span backward d) Digit Span forward e) RAVLT f) HVOT g) RMET h) GDT i) HAMD l) MMSE	PD group made less advantageous selections than HC group, performing lower. There was a significant correlation between the IGT and ToM (r = 0.58, *p* = 0.02).
*Game of Dice Task*					
Brand et al., 2004	Total amount of money earned, Frequency of choosing risky/safe options	a) categorization, set shifting, flexibility b) verbal fluency c) working memory	d) verbal memory e) logical thinking f) visuospatial rotation g) global cognitive functioning	a) MCST (categories, number of perseverative and nonperseverative errors) b) Verbal fluency (phonemic) c) Digit span backward d) Word list e) subtest 4 (*Leistungsprüfsystem*) f) subtest 7 (*Leistungsprüfsystem*) g) MMSE, DemTec	PD group made more risky choices (t = 3.67; *p* = 0.001) and totalized a lower final capital (U = 89.50, *p* = 0.005) in the GDT than the HC group. PD group used less frequently negative feedback for shifting to advantageous decisions, selecting a higher number of risky choices. The frequency of disadvantageous choices in the GDT was correlated with MCST nonperseverative errors (r = 0.51, *p* = 0.021).
Euteneuer et al., 2009	Total amount of money earned, Net score	a) categorization, set shifting, flexibility, b) flexibility, rule monitoring, information processing c) working memory	d) ToM d) verbal ability e) reasoning f) global cognitive functioning g) depression h) DM under ambiguity	a) MCST (categories, number of perseverative and nonperseverative errors) b) Verbal fluencies (phonemic and semantic) c) Digit span backward d) RMET e) Reasoning subtest (*Leistungsprüfsystem*) f) MMSE g) BDI h) IGT	PD group displayed a lower performance than HC group in the GDT (*p* = 0.046), making more risky choices (r = 2.18; *p* = 0.037). In PD group, the GDT net score was correlated with MCST categories (r = 0.51, *p* = 0.018) and perseverative errors (r = −0.50, *p* = 0.021), and with the IGT net score (r = 0.47, *p* = 0.033). The final capital of the GDT was correlated with MCST categories (r = 0.56, *p* = 0.008), perseverative errors (r = −0.45, *p* = 0.042), nonperseverative errors (r = −0.48, *p* = 0.029), and verbal fluencies (r = 0.48, *p* = 0.027).
Xi et al., 2015	Total amount of money earned, Frequency of choosing risky/safe options	a) frontal functions b) inhibitory control c) EFs	d) short-term memory e) verbal memory f) visual perceptual function g) ToM h) DM under ambiguity i)depression l) global cognitive functioning	a) Verbal fluency (semantic) b) Stroop test c) Digit span backward d) Digit Span forward e) RAVLT f) HVOT g) RMET h) IGT i) HAMD l) MMSE	PD group earned a lower total amount of money than HCs, making more risky decisions. A significant correlation emerged between the GDT final capital and the verbal memory delay recall (r = 0.57, *p* = 0.03).

BDI, Beck Depression Inventory; CPT II, Conners’ Continuous Performance Test II; CWIT, Color Word Interference Test; EFs, executive functions; GDT, Game of Dice Task; HAMD, Hamilton Depression Scale; HCs, healthy controls; HVOT, Hooper Visual Organization Task; IGT, Iowa Gambling Task; MCST, Modified Card Sorting Test; MDRS, Mattis Dementia Rating scale; MMSE, Mini-Mental State Examination; PCL, Probabilistic Classification Learning; RAVLT, Rey Auditory Verbal Learning Test; RMET, Reading the Mind in the Eyes Task; SDS, Zung Self-Rating Depression Scale; ToM, Theory of Mind; WCST, Wisconsin Card Sorting Test

Focusing on possible relationships between the decisional task and EFs, significant correlations emerged between the GDT and EFs tests in two of three studies (Brand et al., 2004; Euteneuer et al., 2009). In Brand et al.’ (2004) study, the frequency of risky decisions was related to EFs (specifically, nonperseverative errors in Modified Card Sorting Test; MCST (Nelson, 1976)). The authors divided the PD sample into two subgroups (unimpaired and impaired patients) based on their performance in the assessment of EFs. They found that the group composed of impaired patients made a higher number of risky decisions than the unimpaired patients’ group. No other correlations with verbal fluency or working memory emerged. Likewise, Euteneuer et al. (2009) found correlations between the ability to categorize and shift effectively with both the net score and the total amount earned, and the phonemic fluency with the total earned. Thus, the more PD patients were efficient in categorizing, being flexible, and self-monitoring, the more they made advantageous choices. Otherwise, Xi et al. (2015) did not find correlations between the GDT performance and EFs, but they found a relationship between the decisional performance and the free recall of a word list.

Moreover, concerning possible relationships between DM performance and patients’ clinical features or dopaminergic replacement therapy dosage, only one study explicitly claimed to have investigated them finding no significant results (Euteneuer et al., 2009).

## Discussion

Analyzing in detail the selected studies to shed light on the relationship between DM and EFs in PD patients, the results support the presence of a relationship between EFs and DM performance, which is more evident under conditions of risk (in two of three studies significant correlations emerged between the GDT performance and the abilities to effectively categorize and shift) rather than in conditions of ambiguity (only in Ibarretxe-Bilbao et al.’ (2009) study a relationship between the IGT and working memory was found). It appears that in DM under risk higher-order cognitive processes are required, such as those exploited by EFs, to consider all data before making a choice. Thus, EFs can mainly support DM when the decisional situation is not characterized by a predominant condition of ambiguity, which implies a reduced number of available data to analyze. Referring to the complexity of everyday life, in ambiguous conditions we can assume that EFs (when they are not impaired) could however support, at least in part, the cognitive process of critical analysis regarding which data are missing and how to acquire them, adding more pieces of information to make the condition “less” ambiguous. Further studies can deepen such an issue focusing on decisions in real-life situations.

### Delving into the decisional mechanisms under ambiguity and risk through the Theory of Mind

It seems that EFs can be a resource sustaining the cognitive process underlain DM under risky situations in PD patients. Conversely, it seems that under ambiguous conditions other individual abilities or characteristics may play an important role. These considerations can lead to the assumption that these two decisional conditions can involve different neural circuits, as it was pointed out (Brand et al., 2004; Euteneuer et al., 2009; Xi et al., 2015). In this way, to better disentangle such a difference, results from the studies that investigated the relationship between the IGT and the ToM are worth mentioning (for more details, see Introduction). Three of four studies (Ibarretxe-Bilbao et al., 2009; Mimura et al., 2006; Xi et al., 2015) highlighted moderate positive correlations (Table 4), indicating that the greater PD patients’ functioning of ToM ability was, the fewer risky choices were made under conditions of ambiguity. Such a result can support the possible overlaps of neural areas that are assumed to be exploited by DM under ambiguity and ToM, such as the medial parts of the PFC and BG (Bodden et al., 2013). Conversely, in the two studies which investigated both the ToM and DM under risk (administering the GDT) (Euteneuer et al., 2009; Xi et al., 2015), no correlations were found. Accordingly, it seems to confirm a possible central involvement in DM under predominant ambiguity and in the ToM of crucial structures underlying the orbitofrontal circuit. Whilst under risky conditions, other neural structures may be interested, such as those underling the dorsolateral circuit, which supports EFs functioning.

It appears to be in line with the model proposed by Schiebener and Brand (2015), pointing out that DM, at least under risk, may involve two processes that can interact during a decision since the earlier steps: A process is driven by cognition and the other one by anticipating the emotional reward and punishment. The predominant use of either may depend on decision-maker characteristics (such as individual traits or cognitive functioning) as well as on the decisional condition (such as information available and its salience) (Schiebener and Brand, 2015).

Such a “dichotomous process,” involving abilities that imply emotions and those ones that are “more cognitive,” can be linked to a recent organizing principle that conceptualized EFs as along a continuum from hot EFs to cold EFs, according to the extent that they are emotionally charged. Specifically, hot EFs involve processes connoted by motivation and emotion, such as the incentive value elaboration and the reversal of approach-and-avoidance behaviors, whereas cold EFs underly processes characterized by emotionally neutral situations, involving abilities such as working memory, inhibition, and flexibility (Bechara et al., 1999; Chan et al., 2008; Salehinejad et al., 2021; Zelazo, 2015). Although this conceptualization of EFs is not commonly used and only recent works considered it (Colautti et al., 2022; Damme et al., 2019), it may be useful for a comprehensive and more in-depth analysis of DM processes under ambiguity and risk and the relationship with EFs, considering both the affective aspects involved in reward processing and the cognitive load required by the task.

Furthermore, it appears promising to keep the focus on investigating the possible relationships between cognition (and especially EFs) and the affective aspects (including reward processing) in PD patients also to better understand DM in everyday life situations, which usually occur in a social context and implying direct or indirect consequences for oneself and others (Rilling & Sanfey, 2011). In this respect, moral DM has been recently considered, involving both the ability to infer others’ intentions (and more in general ToM) and cognitive control and EFs (Rosen et al., 2013, 2015). Results showed the presence of differences between HCs and PD patients in cognitive mechanisms related to moral DM. In HCs the decisional performance was related to ToM (Rosen et al., 2013), empathy, and EFs (Rosen et al., 2015), assuming that healthy participants may strategically use such abilities, in particular ToM, facilitating the anticipation and the evaluation of possible consequences implied in choice options. While in PD group no correlation emerged, indicating that patients may not (or present difficult to) integrate these abilities in the decisional process, possibly due to dysfunctions encompassing structures belonging to the corticostriatal circuits (Rosen et al., 2013, 2015), which may result in suboptimal or selfish decisions. Similarly, studies that investigated deceptive DM (a specific type of moral DM; Ponsi et al., 2021) found impaired or reduced deceptive behaviors in patients affected by PD and essential tremor compared with HC groups (Abe et al., 2009; Abe et al., 2018; Mameli et al., 2013). Patients’ displayed performance involved i) difficulties in managing deceptive responses underlain executive dysfunctions, which can be linked to dorsolateral prefrontal circuit functioning (Abe et al., 2009; Mameli et al., 2013), and ii) a reduced motivation to engage in dishonest behaviors, possibly underlain impairment in reward processing where nucleus accumbens may be pivotal (Abe et al., 2018; Ponsi et al., 2021). Such a finding also can be supported by patients affected by movement disorders with ICDs, who generally show the opposite behaviors characterized by a higher tendency in engaging in suboptimal and dishonest decisions, such as lying frequently to hide their pathological behaviors (Brusa et al., 2016; Cilia et al., 2014). Such a tendency may possibly underlie an increased neural response in the nucleus accumbens and more in general in the ventral striatum, which can foster the salience for rewarding stimuli (Abe et al., 2014; Martini et al., 2018; Ponsi et al., 2021). Such findings are consistent with those of the present review, highlighting the presence of a delicate balance in movement disorders between high-order cognition and affective/reward processes supported by cortical and subcortical structures belonging to corticostriatal circuits and affected by the disease.

### Importance of longitudinal studies in studying DM performance and its relationship with EFs

Regarding the possible effects of dopaminergic treatment reported in literature on DM, in the considered studies no direct relationships emerged between the decisional performance and neither the dosage of dopaminergic replacement therapies nor patients’ clinical conditions. A possible explanation may be that the decisional performance can be influenced by the long-term neurobiological or molecular effects of dopamine replacement therapy on reward-processing circuits (Pignatelli & Bonci, 2015; Volkow & Morales, 2015, for neuroplasticity in dopamine circuits). It may be not sufficient to design cross-sectional studies to investigate possible relationships between DM performances and dopaminergic drugs in PD patients or—to have a more comprehensive framework—between DM and EFs and whether and how the dopaminergic therapy can affect such a relationship. In fact, considering the relationship between DM under ambiguity and risk, EFs, and the neural changes that can occur in corticostriatal circuits with the progression of PD, future studies, including longitudinal designs, may be required to explore possible changes in decisional processes and in the relationships with EFs over time. This could be useful to i) take under control the PD samples’ clinical variability, which can undermine possible comparisons and generalizations of the results; ii) observe possible modifications of the relations between DM and EFs over time, bearing in mind that both cognitive efficiency and neural areas involved by PD change throughout the disease progression; iii) delve into the possible role that dopaminergic drugs play in the relationship between DM and EFs, assessing and observing patients over time, also before the first intake of the dopamine replacement therapy (i.e., de novo patients); iv) provide further results that can explain—and maybe confirm—findings that are present in the literature so far (Colautti et al., 2021; Cools et al., 2022; Kjær et al., 2018).

### Limitations

The present study has some limitations. First of all, heterogeneity among the studies was present, even though inclusion/exclusion criteria were designed to reduce it ensuring comparisons. Such heterogeneity may depend on the different study designs, methodologies adopted for collecting and analyzing data, and tests used to assess EFs. Furthermore, clinical features of PD patients varied across the studies (e.g., the duration of the disease was different, possibly underlying different stages of the PD progression, and consequently different subcortical and cortical impairments due to the disease; LEDD also varies across patients of the same study). Thus, we cannot exclude that, at least in part, results may be biased by these clinical features, undermining possible generalizations of the findings. Otherwise, PD patients are generally characterized by higher subjective variability in clinical characteristics, making it difficult to make a strict homogeneous selection of the patients considering all the clinical parameters. Another limitation concerns the low number of studies that investigated the relationships between DM and EFs in PD patients (especially those that administered the GDT), due to the constraint to investigate only the IGT and the GDT. Such a constraint can be seen as another limitation, because the IGT and the GDT are the only tasks that we considered to study this relationship, thus potentially losing information from studies that used other instruments. However, we believe that limiting the focus only to the two most used and reliable tasks to address conditions of ambiguity and risk could facilitate the comparison of studies’ results, by controlling in this way potential differences in behavioral responses that may be due to the different structures and feedback modalities of the several DM tasks (Schiebener and Brand, 2015). To sum up, further studies would be desirable to replicate results increasing the number of studies considering other decisional tasks and to better understand how EFs can support DM in PD patients.

## Conclusions and clinical implications

In the present scoping review, we tried to shed light on the DM mechanisms in PD patients, investigating results in literature concerning the relationship between DM under ambiguity and risk and EFs. Findings suggested that such a relationship is complex and dynamic and EFs seem to have a pivotal role, especially when the cognitive load required by the decisional situation is high, as it happens under situations of risk where more data are available to make a decision. Whilst, when a low number of data are available, it seems that EFs play a minor role. This is in line with the assumption that DM under conditions of ambiguity mainly involves circuits that link BG with OFC and the limbic system (in line with correlations found between the IGT and ToM tests), while DM under risk also underlies the circuit linking BG with dlPFC, which mostly sustains EFs functioning (Brand et al., 2004; Euteneuer et al., 2009; Xi et al., 2015). Thus, EFs can be crucial for supporting DM, at least under risky conditions, in line with Brand et al. (2004), who outlined that PD patients with decreased functioning levels of EFs made suboptimal decisions than patients who did not present such an impairment, underlining the importance of preserving cognition by preventing possible impairments. These results are crucial if we consider the trend toward risky choices displayed by PD patients. Such a tendency is visible both from patients’ difficulties in making representations concerning potential losses and rewards in the long-term along the task (as it appeared in the IGT) and from patients’ preference to choose options characterized by a lower probability to win higher amounts and higher probabilities to lose the same amounts (as it emerged in the GDT). Thus, on one hand, PD patients’ risk-and-reward processing may be biased contributing to making suboptimal and risky choices, but, on the other hand, it is important to keep the focus on the possible role that EFs may play in the decisional process. It may be crucial both to recognize early decisional impairments, which can undermine the quality of life and the therapeutic compliance of the patient (Salvatore et al., 2021), and to better understand the mechanisms implied in suboptimal choices, and consequently to design tailored clinical pathways to sustain the decisional process through the enhancement of EFs involved in DM. This appears even more fundamental considering that people affected by PD can develop selective cognitive impairments up to the initial stages of the disease which can worsen during its progression, representing a risk factor for developing dementia and affecting patients’ autonomy (Saredakis et al., 2019).

## Data Availability

Not applicable.
